# Supplementary material for: Preoperative Risk Stratification of Increased MIB-1 Labeling Index in Pituitary Adenoma: A Newly Proposed Prognostic Scoring System
Source: J Clin Med. 2022 Dec 1;11(23):7151. doi: 10.3390/jcm11237151 (PMC9738462; doi:10.3390/jcm11237151)
Supplement: Supplementary file 1 [file jcm-11-07151-s001.zip › jcm-2009937-supplementary.pdf]

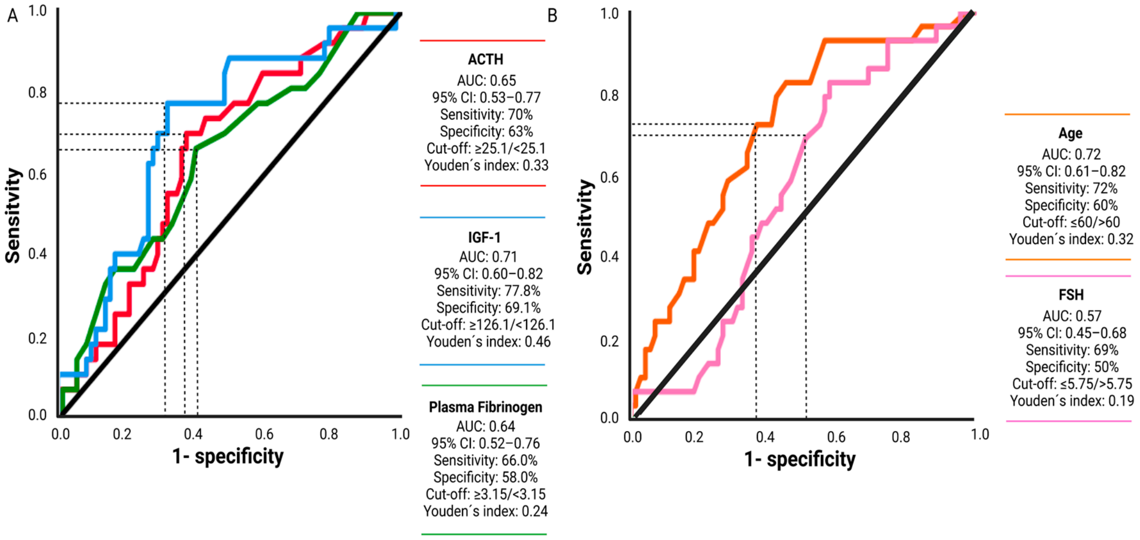

Figure S1. **(A)** Receiver-operating characteristic curve illustrating ACTH, IGF-1, and plasma fibrinogen in the positive correlation with increased MIB-1 labeling index (≥4%) **(B)** Receiver-operating characteristic curve illustrating age and FSH in the inverse correlation with increased MIB-1 labeling index (≥4%).
